# Pelvic Muscle Rehabilitation: A Standardized Protocol for Pelvic Floor Dysfunction

**DOI:** 10.1155/2014/487436

**Published:** 2014-06-11

**Authors:** Rodrigo Pedraza, Javier Nieto, Sergio Ibarra, Eric M. Haas

**Affiliations:** ^1^Division of Minimally Invasive Colon and Rectal Surgery, Department of Surgery, The University of Texas Medical School at Houston, Houston, TX 77054, USA; ^2^Pelvic Health and Physical Therapy Center, Houston, TX 77054, USA

## Abstract

*Introduction*. Pelvic floor dysfunction syndromes present with voiding, sexual, and anorectal disturbances, which may be associated with one another, resulting in complex presentation. Thus, an integrated diagnosis and management approach may be required. Pelvic muscle rehabilitation (PMR) is a noninvasive modality involving cognitive reeducation, modification, and retraining of the pelvic floor and associated musculature. We describe our standardized PMR protocol for the management of pelvic floor dysfunction syndromes. *Pelvic Muscle Rehabilitation Program*. The diagnostic assessment includes electromyography and manometry analyzed in 4 phases: (1) *initial baseline phase*; (2) *rapid contraction phase*; (3) *tonic contraction and endurance phase*; and (4) *late baseline phase*. This evaluation is performed at the onset of every session. PMR management consists of 6 possible therapeutic modalities, employed depending on the diagnostic evaluation: (1) *down-training*; (2) *accessory muscle isolation*; (3) *discrimination training*; (4) *muscle strengthening*; (5) *endurance training*; and (6) *electrical stimulation*. Eight to ten sessions are performed at one-week intervals with integration of home exercises and lifestyle modifications. *Conclusions*. The PMR protocol offers a standardized approach to diagnose and manage pelvic floor dysfunction syndromes with potential advantages over traditional biofeedback, involving additional interventions and a continuous pelvic floor assessment with management modifications over the clinical course.

## 1. Introduction

Pelvic floor dysfunction syndromes include voiding, sexual, and anorectal disturbances characterized by urinary and fecal incontinence, pelvic pain, and/or constipation/anismus. Many patients with such syndromes present with concomitant pelvic functional disorders resulting in complex clinical picture [1]. As such, a modern treatment algorithm involving the concept of an integrated pelvic unit rather than treatment based on isolated organ dysfunction serves to optimize clinical outcomes [2].

Pelvic muscle rehabilitation (PMR) is a multidisciplinary program that involves numerous rehabilitation principles such as muscle floor retraining, biofeedback, and electrical stimulation of the pelvic floor and functionally associated musculature. This program integrates the clinical presentation and assessment of the pelvic floor to identify the physiologic stressors and abnormalities from which a therapeutic program is individualized and prescribed. We initially introduced the concept into our practice in 2006 and we have since optimized the program based on clinical outcomes and reproducibility of results.

We present and describe the basis of our comprehensive PMR protocol for the diagnosis and management of pelvic floor dysfunction syndromes. The protocol encompasses the concepts of muscle floor training, physiologic quieting, biofeedback, electrical stimulation, and discrimination training.

## 2. Pelvic Muscle Rehabilitation Program

### 2.1. Diagnostic Evaluation

Patients presenting with a prior diagnosis of pelvic floor dysfunction including urinary incontinence (UI), fecal incontinence (FI), obstructed defecation syndrome (ODS), and chronic pelvic pain (CPP) undergo a thorough evaluation, which initiates with an interview and review of symptoms. The patient interview is focused on the onset and severity of symptoms, past medical and obstetric history, medications, and history of psychological or social stressors. A systematic review of symptoms is performed to identify the presence of contributing factors such as gastrointestinal, genitourinary, endocrine, or pelvic floor disorders. Depending on the presentation, a series of pelvic health-related or bowel-focused questionnaires are completed by each patient to assess overall quality of life and severity of dysfunction.

A physical exam is then performed including a thorough evaluation of the pelvic floor functional anatomy. Diagnostic evaluations and treatments are done using the Pathway CTS 2000 (The Prometheus Group, Dover, New Hampshire, USA) and are performed to assess the physiologic and functional status of the pelvic floor and accessory muscle groups. These tests include anal (or vaginal) manometry and electromyography (EMG). Manometry is performed to quantify muscle tone and contractility of pelvic muscles using a pressure sensor inserted through the anal sphincter. On verbal command, the patient is asked to voluntarily contract and relax the anal sphincter muscles. The series of contractions and relaxations are repeated and the results are recorded over a specific time interval. Baseline manometric results can identify altered pelvic muscle function and categorize the pelvic floor syndrome into two broad categories: hypotonic and hypertonic.

Electromyography is performed using an internal vaginal or rectal sensor and surface patch electrodes to evaluate accessory muscle activity. Two EMG surface electrodes are placed on the rectus abdominal muscle, two fingerbreadths apart and medial to the anterior superior iliac spine (ASIS), and one ground electrode is placed on the hipbone. With the internal sensor inserted, the patient is asked to repetitively contract and relax the pelvic floor muscles. Measurements are recorded and analyzed in 4 phases: (1)* initial baseline phase:* 60-second evaluation with the patient at rest to determine the initial resting baseline EMG; (2)* rapid contraction phase:* recording of electrical activity while performing five phasic rapid contractions; (3)* tonic contraction and endurance phase:* recording of electrical activity of pelvic floor and abdominal wall muscles following a total of 5 contractions of 10 seconds each, with a resting interval time of 10 seconds; (4)* late baseline phase:* 60-second evaluation with the patient at rest to determine the final resting baseline EMG activity.

### 2.2. Therapeutic Intervention

Following the diagnostic evaluation, the practitioner prescribes a therapeutic program in which one or a combination of 6 modalities is involved. The therapeutic sessions are guided by the patient clinical presentation and the results of the diagnostic EMG and/or manometry. Each session will last from 15 to 45 minutes depending on the modalities utilized and the response to the therapy. The combination of the diagnostic assessment and therapeutic intervention is what we call PMR.

**Figure 1:**
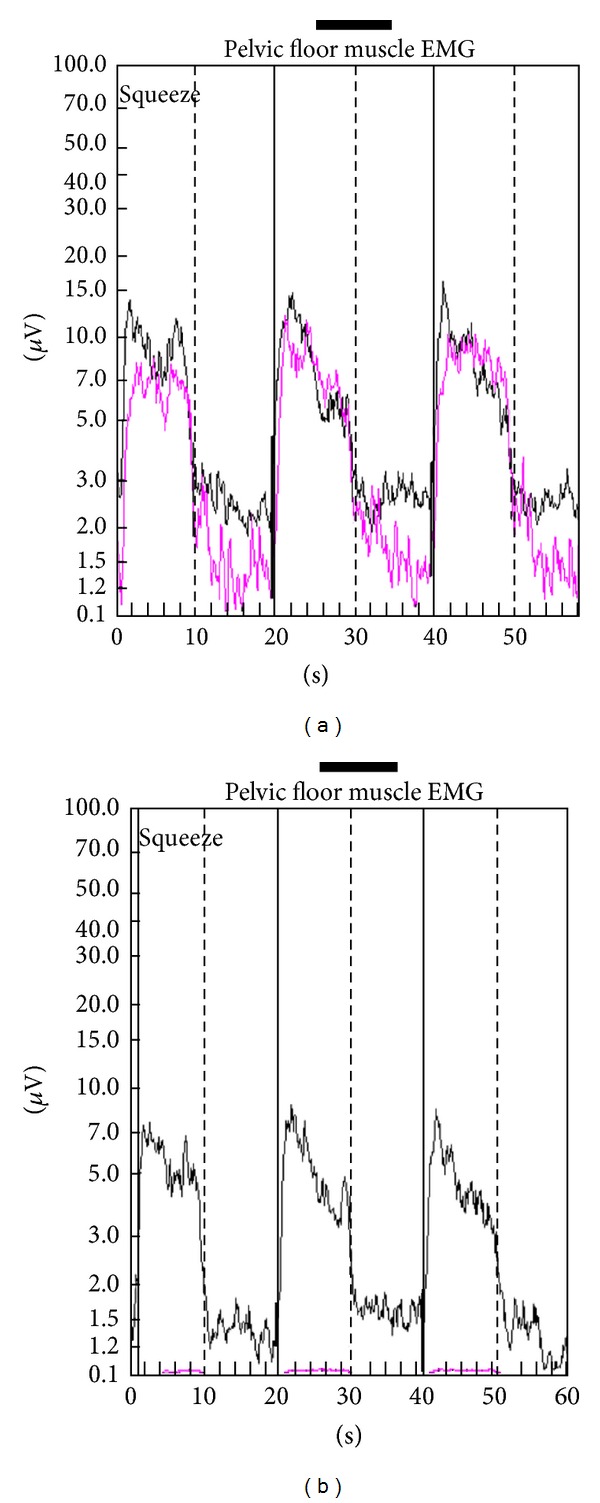
Muscle isolation. (a) Electromyographic tracings of a patient with outlet obstruction defecation and anismus with excessive accessory muscle utilization (pink tracing) corresponding to contraction and relaxation of the pelvic floor complex. (b) Electromyography of the same patient following sessions of pelvic muscle rehabilitation revealing significant reduction of accessory muscle utilization during pelvic floor muscle contraction.

**Figure 2:**
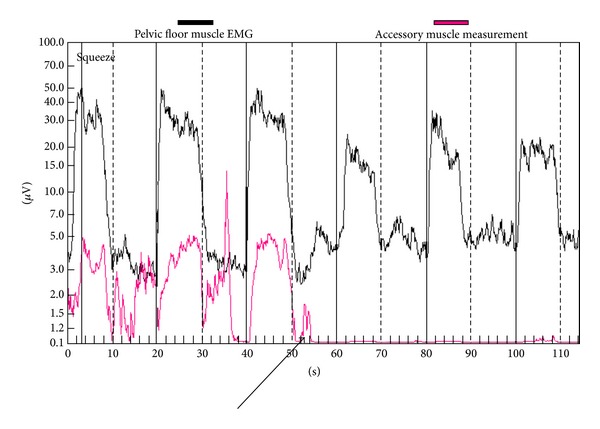
Discrimination training. The electromyographic tracings of a patient with postprostatectomy urinary incontinence showing accessory muscle hyperactivity (pink) during pelvic floor muscle contractions. The accessory muscle electrical activity is entirely suppressed following successful patient sensory awareness training (arrow).

**Figure 3:**
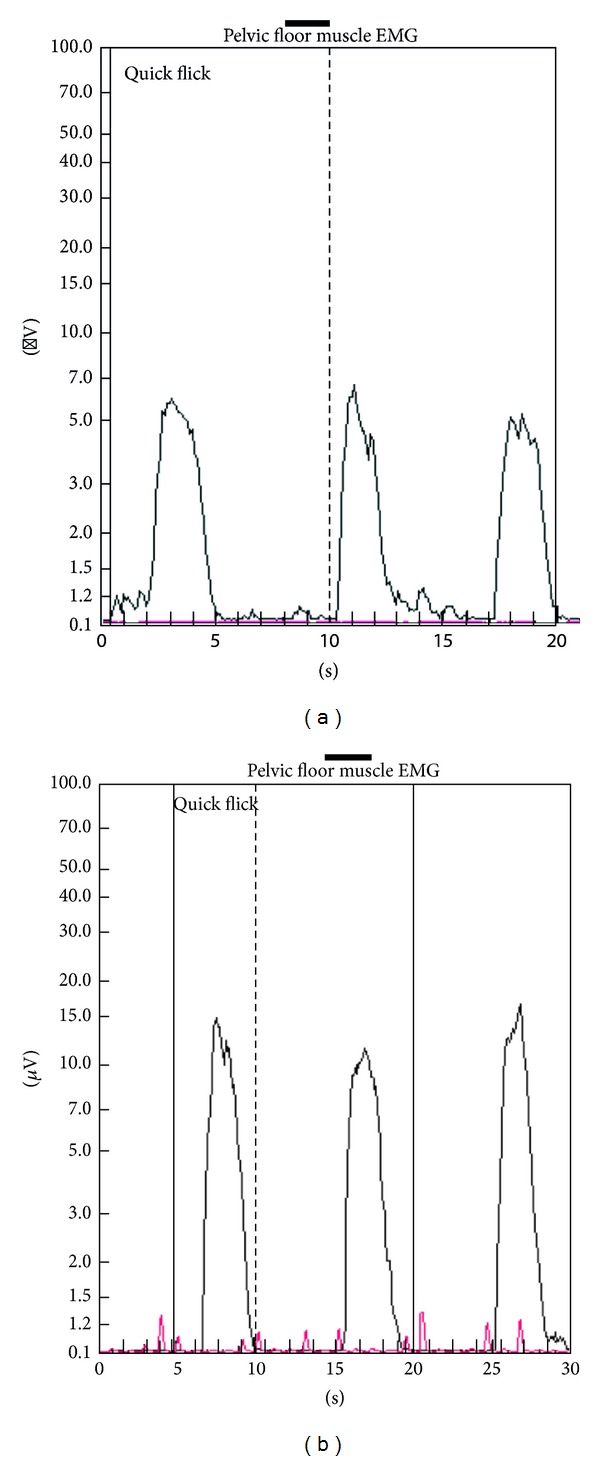
Pelvic floor muscle strengthening. (a) Electromyographic tracing of a patient with stress urinary incontinence during a series of rapid contractions (“quick flicks”) in which the patient is asked to contract and relax over multiple 5-second intervals. The tracing reveals reduced electrical activity and recruitment during contractions (5 mcV) on the initial session. (b) Same patient following sessions of pelvic muscle rehabilitation revealing marked increased electromyographic activity during the contraction (15 mcV) phase of the rapid contraction (“quick flicks”).

**Figure 4:**
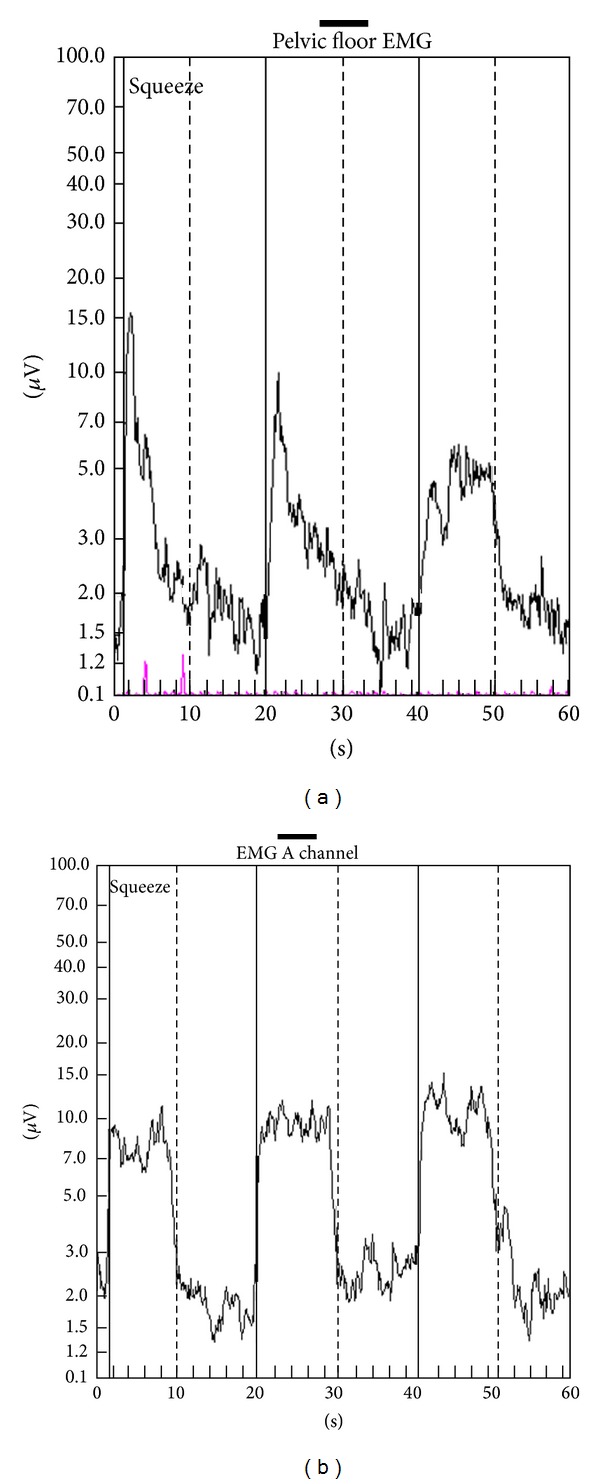
Endurance training. (a) Electromyographic tracings of a patient with fecal incontinence (black tracing) showing inability to maintain sustained contractions (poor endurance) and characteristic sawtooth pattern indicating fatigue. (b) Electromyographic tracings of the same patient following pelvic muscle rehabilitation sessions revealing sustained contractions during each interval contraction.

**Figure 5:**
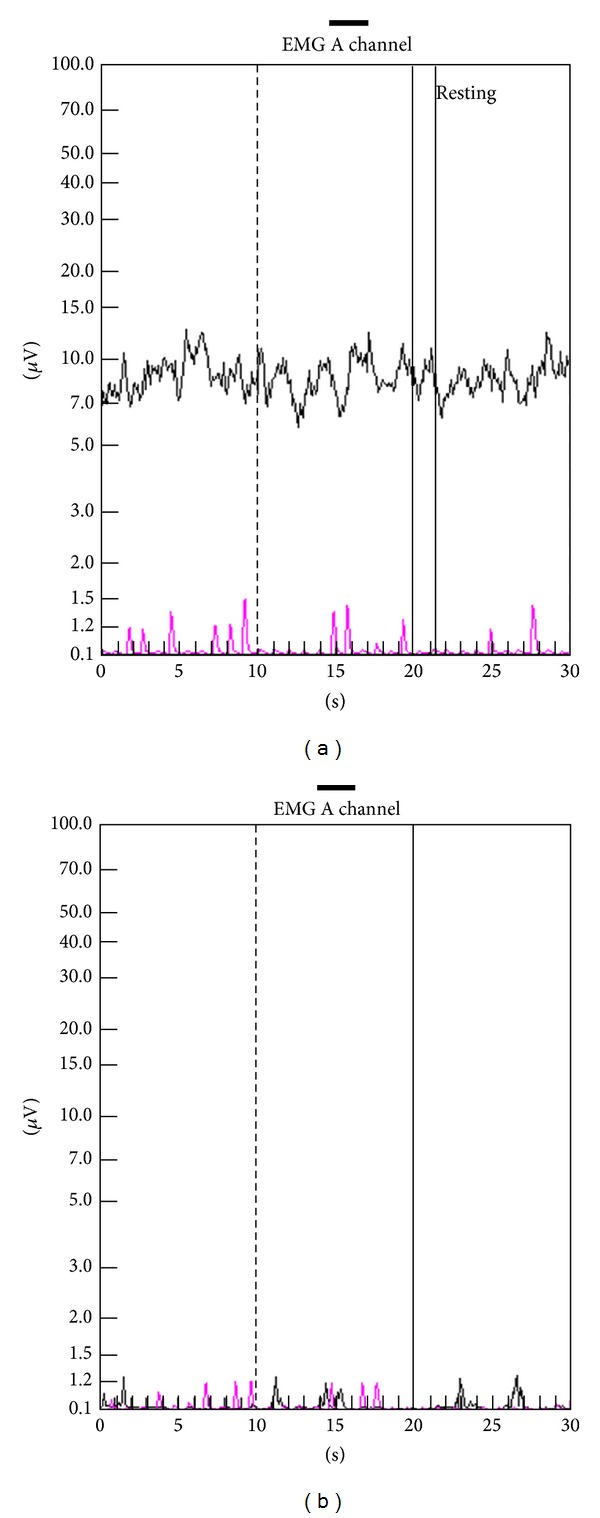
Down-training. (a) Electromyographic tracing of a patient with chronic pelvic pain due to pelvic floor muscle spasm revealing elevated resting tone. (b) Same patient of (a): following 8 sessions of pelvic muscle rehabilitation, the resting muscle tone and symptoms decreased substantially.

**Figure 6:**
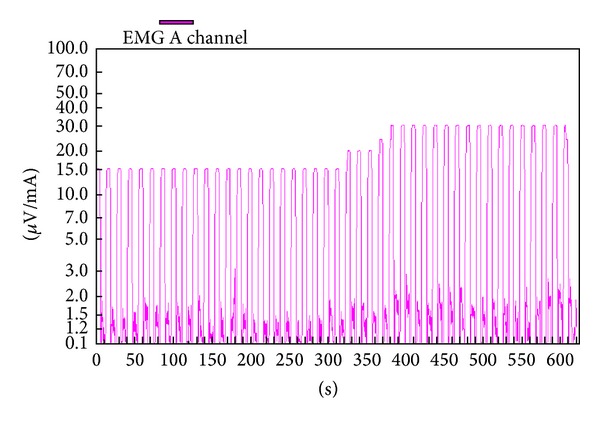
Electrical stimulation. Tracing of a patient with urge urinary incontinence associated with pelvic floor spams. Electrical stimulation mode serves to desensitize muscles to graded elevation of muscle stimuli. With the baseline set to 15 mcV/mA, a baseline electromyographic pelvic floor activity is seen. As the stimulation mode increases to 30 mcV/mA, the electrical activity does not increase, as it is desensitized.

The PMR therapeutic modalities are derived and tailored to the individual patient based on results and findings of the diagnostic pelvic floor muscle evaluation. These modalities included the utilization of one or a combination of six possible therapeutic modalities. The modalities include the following: (1)* muscle isolation:* elimination of accessory muscle substitution through identification and modulation of associated muscle groups (Figure 1); (2)* discrimination training:* enhancement of sensory awareness of tension and release variations to maximize conscious control of muscle contraction (Figure 2); (3)* pelvic floor muscle strengthening:* enhancement of PF muscle motor recruitment (Figure 3); (4)* endurance training:* maintenance of isolated PF muscle motor recruitment by sustained contractions (Figure 4); (5)* down-training:* inhibition of hypertonic muscle activity to lower elevated resting tone (Figure 5); and (6)* electrical stimulation:* employed as the final therapeutic approach to facilitate muscle motor recruitment and controlled fatigue (Figure 6). Each of these modalities is offered in various combinations during each visit according to the clinical and physiological presentation defined during the history and diagnostic assessment.

Additional adjunct therapies offered for patients with CPP, UI, and ODS associated with levator and pelvic floor spasm include physical therapy and cognitive behavioral therapy. Physical therapy encompasses the manual manipulation of the pelvic floor and includes visceral and muscular manipulation as well as bone alignment and trigger point release. Cognitive behavioral therapy consists of various psychotherapy sessions in an individual or couple setting and relies heavily on a well-developed patient-therapist relationship. This goal-oriented technique addresses dysfunctional emotions, behaviors, and cognitive processes related to the patient's pelvic disorder.

## 3. Discussion

We describe a standardized PMR protocol for the assessment and management of pelvic floor dysfunction syndromes. The protocol is employed not only to address a single manifestation (e.g., urinary incontinence) but also to treat complex presentations and those with multiple disorders (e.g., urinary incontinence and fecal incontinence). This PMR protocol involves the principles of pelvic floor and associated muscle groups isolation, discrimination, strengthening, and conditioning. Furthermore, the concepts of biofeedback, physiological quieting, and electrical stimulation are utilized as an adjunct to achieve functional and clinical enhancement of pelvic floor dysfunction syndromes.

Traditional pelvic floor treatment protocols typically involve a single modality of intervention such as biofeedback or muscle training or a combination of two modalities. Such protocols have been employed with variable rates of success. It has been shown that the addition of biofeedback to muscle exercise programs results in improved fecal incontinence outcomes compared with muscle exercises alone [3]. For urinary incontinence, pelvic muscle exercises with biofeedback result in increased strength and improved patient satisfaction in comparison to pelvic muscle exercise training alone [4, 5]. Based on these data and clinical observations, our program includes the utilization of a combination of these and additional techniques to promote clinical improvement in those presenting with pelvic floor muscle dysfunction syndrome.

Our approach with a comprehensive PMR program differs from standard biofeedback in many ways. One of the unique features of the PMR protocol is that it is heavily dependent on the treating practitioner and the therapeutic interventions are both individualized and adjusted at each session based on the clinical and physiological picture. The practitioner defines and identifies the pelvic floor physiologic disturbances through diagnostic testing (i.e., EMG and manometry) and then establishes the therapeutic modalities that are best employed to overcome the abnormalities in a step-wise fashion. Thus, it is critical that the rehabilitation sessions begin with a thorough discussion of the clinical symptoms combined with an assessment of the pelvic floor functional and physiological abnormalities.

Another difference is that in traditional biofeedback program, the patient is prescribed a protocol and the same program is performed at given intervals to achieve pelvic retraining. The limitation of such a program is that it is a defined and fixed protocol and does not vary or take into consideration response to therapy or alterations in pelvic physiology. Furthermore, the patients are often dependent on the visual cues of the biofeedback as a crutch to achieve the functional goal of the program. Hence, the effective and successful cognitive training of the pelvic floor muscle complex is often not translated into activities of daily living in which these sensory cues are absent. In our PMR protocol, the patients are reevaluated and rediagnosed on each visit to determine the proper intervention at the given visit. As such, the therapeutic modalities employed on each visit may vary from the previous or further interventions. This approach affords continuing assessment of the functional and clinical pelvic floor dysfunction appropriately addressing their management to achieve best results.

**Figure 7:**
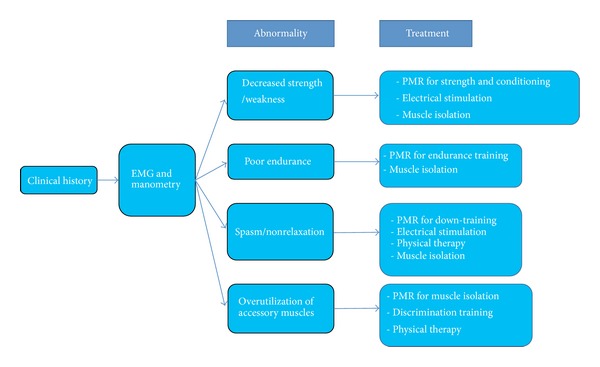
Algorithm illustrating the diagnostic decision tree and treatment approach. Reassessment is performed every time the patient returns for the next session.

The primary single endpoint of our protocol is to improve quality of life. The protocol relies on patient satisfaction Likert scale and the goal is set for good or excellent improvement. Nonetheless, physiologic parameters are evaluated on each visit to document progress and serve also as endpoints of the protocol. We consider the clinical presentation along with interpretation of the physiologic abnormalities identified on EMG/manometry to direct the therapeutic intervention on a case by case basis (Figure 7). From this perspective, depending on the abnormality, we continue the treatment until it is overcome. For instance, if we identify severe pelvic floor muscle spasm in a patient with chronic pelvic pain, we initiate a PMR program directed at “down-training” which in essence aims to relax and/or fatigue the pelvic floor muscles. If we identify significantly diminished strength and poor endurance in a patient with fecal incontinence, we utilize a PMR protocol for muscle strengthening along with endurance training to overcome the functional abnormalities. If within two successive sessions there is no improvement in both clinical and physiologic evaluations, we classify the case as failure, and the pelvic muscle rehabilitation program is cancelled.

In our experience, we believe there is a positive concordance rate between clinical outcomes and physiologic parameters on EMG and manometry. However, the extent and reproducibility of these correlations in regard to quality of life metrics will require further investigation.

Although an economic analysis was beyond the scope of this paper, an important consideration of any protocol is the associated costs and fees. Such a cost analysis will need to include the fees associated with surgical interventions and other resources that are often avoided following completion of a pelvic muscle rehabilitation program.

## 4. Conclusion

The PMR protocol offers a standardized and comprehensive approach to both diagnose and manage pelvic floor dysfunction syndromes. This PMR program provides potential advantages over traditional biofeedback, as it involves the employment of additional therapeutic interventions including muscle isolation, discrimination, conditioning, and strengthening, as well as physiological quieting and electrical stimulation. Moreover, the PMR program affords a continuous pelvic floor assessment with the according management modifications. Further clinical studies are warranted to evaluate the clinical results of the PMR protocol to formulate solid conclusions.
